# Supplementary material for: Dietary Fat Intake among Chinese Adults and Their Relationships with Blood Lipids: Findings from China Nutrition and Health Surveillance and Comparison with the PURE Study
Source: Nutrients. 2022 Dec 9;14(24):5262. doi: 10.3390/nu14245262 (PMC9788542; doi:10.3390/nu14245262)
Supplement: Supplementary file 1 [file nutrients-14-05262-s001.zip › nutrients-2063881-supplementary.pdf]

File S1. Definitions of food groups and nutrients, and methods for calculating intake.

In present study, we classified 2270 food items in the constantly updating food composition database into 12 food groups, including pork, other meats, processed meats, poultry, fish, dairy and eggs, nuts and soybeans, oils of high monounsaturated fatty acids (MUFA), oils of high polyunsaturated fatty acids (PUFA), MUFA-PUFA balanced oils, oils of high saturated fatty acids (SFA), and other foods. Definition of four types of cooking oils and processed food see Table S1.

Table S1. Definitions of selected food groups.

Food category	Definition
Processed meat	Meat that has been transformed through salting, curing, fermentation, smoking, or other processes to enhance flavor or improve preservation, particularly those industrially processed [38].
High-MUFA oils	Oils with the proportion of monounsaturated fatty acids exceeding 50 percent of total fat content, such as rapeseed oil, tea-seed oil and olive oil.
MU-PUFA balanced oils	Oils with the proportions of monounsaturated and polyunsaturated fatty acids both ranging between 30 and 50 percent of total fat content, such as sesame oil, salad oil and peanut oil.
High-PUFA oils	Oils with the proportions of polyunsaturated fatty acids exceeding 50 percent of total fat content, such as soybean oil, corn oil, sunflower oil and walnut oil.
High-SFA oils	Fats with the proportions of saturated fatty acids exceeding 40 percent of total fat content, such as lard, butter and coconut oil.

Abbreviation: MUFA, monounsaturated fatty acids; PUFA, polyunsaturated fatty acids; SFA, saturated fatty acids.

Table S2. Characteristics of participants according to quintiles of energy-adjusted total fat and fats of various types and sources (n = 39,115).

	Total fat			Animal fat			SFA			MUFA			PUFA		
	**Q1**	**Q3**	**Q5**	**Q1**	**Q3**	**Q5**	**Q1**	**Q3**	**Q5**	**Q1**	**Q3**	**Q5**	**Q1**	**Q3**	**Q5**
Total energy intake *, kcal	2005.9 ± 565.5	1571.0 ± 512.2	1938.6 ± 607.5	2082.9 ± 538.8	1546.2 ± 524	1905.2 ± 594.6	2040.8 ± 559.6	1550.1 ± 514.7	1904.6 ± 601.3	2038.1 ± 561.5	1564.6 ± 510.3	1896.7 ± 603.8	2075.4 ± 567.7	1568.9 ± 503.1	1865.9 ± 606.9
Carbohydrate, E%	67.5 ± 8.3	53.3 ± 5.3	36.4 ± 7.0	57.5 ± 11.4	53.9 ± 10.8	42.6 ± 9.7	64.5 ± 10.6	52.9 ± 8.6	40.1 ± 9.2	65.6 ± 9.3	52.7 ± 8.1	39.3 ± 9.1	59.7 ± 12.8	53.3 ± 10.8	44.3 ± 11
Protein, E%	12.5 ± 2.7	13.0 ± 3.5	10.9 ± 3.5	11.9 ± 3.0	12.8 ± 3.2	12.9 ± 3.8	11.4 ± 2.7	12.8 ± 3.4	12.5 ± 3.9	12.5 ± 2.8	13.2 ± 3.5	10.7 ± 3.4	12.7 ± 3.2	12.9 ± 3.4	10.9 ± 3.3
Total fat *, E%	20.1 ± 5.5	34.5 ± 2.5	53.4 ± 7.3	31.5 ± 11.7	34.2 ± 10.9	44.9 ± 9.9	24.4 ± 10	35.1 ± 8.8	47.9 ± 9.7	22.1 ± 7.4	35.0 ± 7.1	50.5 ± 9.1	27.5 ± 11.5	34.7 ± 10	45.4 ± 11.3
Animal fat *, E%	7.0 ± 5.9	13.9 ± 8.6	22.2 ± 15	6.4 ± 3.4	11.4 ± 2.8	32.1 ± 9.1	4.4 ± 4.0	11.9 ± 5.7	29.7 ± 11.7	6.8 ± 5.6	14.6 ± 8.7	20.5 ± 15.1	17.9 ± 12.8	14.3 ± 11.1	9.9 ± 8.0
Plant fat *, E%	13.0 ± 5.8	20.4 ± 8.6	31 ± 16.1	24.9 ± 11.7	22.6 ± 11	12.7 ± 9.1	19.9 ± 10.5	23.0 ± 11.4	18.1 ± 13.8	15.2 ± 7.8	20.2 ± 10.3	29.9 ± 14.7	9.5 ± 6.3	20.2 ± 6.6	35.3 ± 11.1
SFA *, E%	4.8 ± 1.9	8.0 ± 2.4	12.0 ± 4.6	6.1 ± 2.1	7.4 ± 2.0	13.3 ± 3.8	4.1 ± 1.3	7.5 ± 0.8	13.9 ± 3.4	5.1 ± 2.0	8.4 ± 2.6	10.7 ± 5.0	8.2 ± 4.8	7.9 ± 3.7	8.7 ± 2.9
Animal SFA *, E%	2.6 ± 2.1	4.9 ± 3.1	7.8 ± 5.8	2.3 ± 1.3	4.0 ± 1.2	11.3 ± 4.1	1.5 ± 1.3	4.1 ± 1.8	10.8 ± 4.6	2.5 ± 2.1	5.1 ± 3.2	7.3 ± 5.7	6.5 ± 4.9	5.0 ± 4.0	3.4 ± 2.7
Plant SFA *, E%	2.2 ± 1.0	3.1 ± 1.5	4.1 ± 2.5	3.7 ± 1.8	3.4 ± 1.7	2.0 ± 1.4	2.6 ± 1.0	3.4 ± 1.6	3.1 ± 2.5	2.6 ± 1.2	3.3 ± 1.8	3.5 ± 2.2	1.7 ± 1.3	2.9 ± 1.0	5.2 ± 1.8
MUFA *, E%	7.3 ± 2.9	13.2 ± 3.3	22.2 ± 6.5	12.0 ± 6.6	13.3 ± 6.3	18.1 ± 5.2	10.2 ± 6.7	13.4 ± 6.0	18.9 ± 5.1	6.7 ± 2.3	12.7 ± 1.4	24.0 ± 5.1	11.3 ± 5.4	14.4 ± 5.9	15.4 ± 7.8
Animal MUFA *, E%	2.6 ± 2.4	5.3 ± 3.5	8.8 ± 6.4	2.3 ± 1.3	4.2 ± 1.6	12.8 ± 4.3	1.6 ± 1.5	4.4 ± 2.3	11.9 ± 5.2	2.4 ± 2.1	5.5 ± 3.4	8.3 ± 6.6	7.0 ± 5.3	5.5 ± 4.6	3.7 ± 3.4
Plant MUFA *, E%	4.6 ± 2.9	7.9 ± 4.5	13.4 ± 8.7	9.7 ± 6.5	9.0 ± 6.1	5.3 ± 4.8	8.6 ± 6.8	9.0 ± 6.3	7.1 ± 5.9	4.2 ± 2.1	7.2 ± 3.4	15.7 ± 8.5	4.3 ± 4.0	8.9 ± 5.0	11.7 ± 7.4
PUFA *, E%	5.7 ± 2.8	9.2 ± 4.0	13.3 ± 6.7	10.4 ± 5.6	9.7 ± 5.1	7.1 ± 3.4	7.6 ± 4.1	10.1 ± 5.2	9.2 ± 5.5	7.8 ± 4.8	9.6 ± 5.5	10.5 ± 4.4	4.1 ± 1.2	8.2 ± 0.8	17.3 ± 4.9
Dietary fiber [a,*], g	11.1 ± 6.6	9.8 ± 5.6	7.4 ± 5.7	10.5 ± 7.3	9.8 ± 5.2	8.0 ± 6.2	10.9 ± 7.4	9.7 ± 5.2	7.6 ± 5.6	11.4 ± 7.6	9.6 ± 4.7	7.6 ± 6.2	9.7 ± 7.6	9.8 ± 5	8.7 ± 5.9
Dietary cholesterol [a], g	0.2 ± 3.8	0.2 ± 3.0	0.2 ± 6.2	0.1 ± 2.6	0.3 ± 5.9	0.1 ± 3.4	0.2 ± 3.9 †	0.2 ± 2.9 †	0.1 ± 2.9 †	0.3 ± 4.7 *	0.2 ± 2.9 *	0.1 ± 2.3 *	0.2 ± 4.2	0.2 ± 3.2	0.2 ± 6
Vegetables [a,*], g	229.6 ± 155.8	242.9 ± 131.6	236.2 ± 143.7	218.5 ± 155.5	237.3 ± 131.5	262.9 ± 142.6	221.1 ± 153.6	243.9 ± 129.0	248.0 ± 145.5	226.3 ± 157.6	242.3 ± 130.9	242.5 ± 141.8	247.5 ± 162.5	246.4 ± 131.4	216.4 ± 136.8
Fruits [a], g	32.9 ± 74.3 *	38.2 ± 67.2 *	24.3 ± 56.0 *	28.6 ± 69.9 †	39.5 ± 68.7 †	27.0 ± 59.6 †	27.0 ± 67.3 †	38.4 ± 66.5 †	28.6 ± 61.5 †	40.0 ± 82.7 *	37.3 ± 64.3 *	19.4 ± 48.4 *	30.1 ± 73.6 *	33.4 ± 61.6 *	36.1 ± 69 *
Age, year	51.7 ± 10 *	50.9 ± 10.4 *	51.2 ± 10 *	52.1 ± 9.8 *	51.2 ± 10.2 *	50.1 ± 10.3 *	52.1 ± 9.9 *	51.2 ± 10.4 *	50.2 ± 10.3 *	51.4 ± 10 †	51.0 ± 10.4 †	51.5 ± 10 †	50.8 ± 10.2 *	51.1 ± 10.4 *	51.5 ± 9.9 *
Male *, %	54.1	40.3	47.1	53.8	39.0	49.4	54.1	39.5	47.3	54.6	39.8	46.6	54.3	39.7	46.3
Urban location, %	30.4 *	42.5 *	36.7 *	28.7 *	44.7 *	34.9 *	27.2 *	44.0 *	37.5 *	32.7 †	43.7 †	34.4 †	28.3 *	42.8 *	38.3 *
South *, %	42.7	60.9	79.1	39.2	59.5	86.9	41.8	60.4	82.9	33.9	61.3	86.0	69.5	63.7	46.4
Illiteracy, %	14.7 †	12.9 †	12.8 †	16.1 *	12.4 *	11.5 *	17.3 *	12.0 *	11.8 *	13.0 *	12.2 *	15.1 *	15.3 *	12.5 *	12.4 *
Current smoker, %	30.2 †	24.3 †	29.1 †	29.0 *	23.9 *	31.9 *	29.7 †	23.7 †	29.6 †	30.1 †	25.0 †	28.6 †	32.9 *	23.2 *	27.6 *
Excessive drinker, %	15.3 *	11.7 *	13.9 *	14.2 †	11.5 †	14.6 †	14.1 †	11.1 †	13.6 †	15.5 *	11.4 *	13.8 *	16.4 *	11.3 *	13.4 *
Physical activity, MET-h/week	27.3 ± 25.1 *	23.6 ± 22.6 *	26.2 ± 24.3 *	27.0 ± 24.7 †	23.5 ± 22.6 †	27.7 ± 25.3 †	27.9 ± 25.1 *	23.2 ± 22.1 *	26.7 ± 24.8 *	26.7 ± 24.8 *	22.9 ± 22.2 *	27.5 ± 24.7 *	29.0 ± 25.9 *	23.8 ± 22.6 *	24.4 ± 23.5 *
BMI *, kg/m^2	24.3 ± 3.4	24.1 ± 3.4	24.0 ± 3.4	24.4 ± 3.5	24.2 ± 3.4	23.6 ± 3.3	24.2 ± 3.4	24.2 ± 3.4	23.7 ± 3.4	24.5 ± 3.4	24.2 ± 3.5	23.8 ± 3.3	23.9 ± 3.4	24.1 ± 3.5	24.5 ± 3.5
Hypertension *, %	41.5	37.7	39.6	43.2	39.5	35.6	42.9	39.2	36.2	41.9	38.2	39.5	38.1	38.7	42.1
Family history *, %	38.8	40.0	33.8	38.1	41.9	31.8	36.9	40.8	32.0	41.3	39.9	32.1	33.0	39.2	41.2

* $p_{\text{-trend}} < 0.05$; when marked in right upper corner of variable names, it means significant linear trends presented across quintiles of all fat variables; otherwise, the linear trend presented only across the quintiles of corresponding fat variable. † $p < 0.05$ for between/among-group comparison; the position of this symbol means the same as above. [a] Energy adjusted. Abbreviation: MET, metabolic equivalents of task; BMI, body mass index.

Table S3. Comparison of the proportion of various fats in total fat intake, stratified by the median of total fat (n = 39,115).

	**<Median, %**	**≥Median, %**	***p* value**
SFA	23.7	22.9	<0.0001
Animal SFA	13.1	14.7	<0.0001
Plant SFA	10.4	8.2	<0.0001
MUFA	36.7	40.1	<0.0001
Animal MUFA	13.5	16.3	<0.0001
Plant MUFA	23.0	23.6	<0.0001
PUFA	28.0	25.4	<0.0001
Animal PUFA	3.2	3.6	<0.0001
Plant PUFA	24.7	21.7	<0.0001

Table S4. Regression coefficient (95% CI) and standardized coefficients for the association between dietary fats (per 11 g) and lipid markers *.

Fats	Lipid markers	β (95% CI)	Standard β (95% CI)	*p* value
Total fat	TC, mmol/L	0.010 (0.006–0.014)	0.035 (0.021–0.049)	<0.0001
	LDL-c, mmol/L	0.006 (0.002–0.010)	0.024 (0.008–0.04)	0.001
	HDL-c, mmol/L	0.004 (0.002–0.006)	0.039 (0.02–0.058)	<0.0001
	TG, mmol/L	−0.005 (−0.009–−0.001)	−0.015 (−0.027–−0.003)	0.057
	TCHDL	−0.004 (−0.008–−0.000)	−0.012 (−0.023–0.000)	0.136
	TGHDL	−0.009 (−0.015–−0.003)	−0.021 (−0.034–−0.007)	0.006
SFA	TC, mmol/L	0.120 (0.106–0.134)	0.119 (0.106–0.133)	<0.0001
	LDL-c, mmol/L	0.098 (0.086–0.110)	0.111 (0.097–0.124)	<0.0001
	HDL-c, mmol/L	0.013 (0.009–0.017)	0.036 (0.025–0.047)	<0.0001
	TG, mmol/L	0.009 (−0.007–0.025)	0.008 (−0.006–0.021)	0.222
	TCHDL	0.057 (0.041–0.077)	0.047 (0.034–0.061)	<0.0001
	TGHDL	−0.005 (−0.025–0.015)	−0.003 (−0.016–0.01)	0.642
Animal SFA	TC, mmol/L	0.112 (0.100–0.124)	0.106 (0.095–0.117)	<0.0001
	LDL-c, mmol/L	0.089 (0.079–0.099)	0.096 (0.085–0.106)	<0.0001
	HDL-c, mmol/L	0.010 (0.006–0.014)	0.026 (0.016–0.037)	<0.0001
	TG, mmol/L	0.041 (0.027–0.055)	0.033 (0.022–0.044)	<0.0001
	TCHDL	0.062 (0.048–0.076)	0.049 (0.038–0.06)	<0.0001
	TGHDL	0.029 (0.011–0.047)	0.018 (0.007–0.029)	0.002
Plant SFA	TC, mmol/L	−0.047 (−0.072–−0.022)	−0.02 (−0.03–−0.009)	0.000
	LDL-c, mmol/L	−0.028 (−0.052–−0.004)	−0.013 (−0.025–−0.002)	0.019
	HDL-c, mmol/L	0.008 (−0.002–0.018)	0.009 (−0.002–0.021)	0.096
	TG, mmol/L	−0.16 (−0.192–−0.129)	−0.057 (−0.068–−0.046)	<0.0001
	TCHDL	−0.067 (−0.096–−0.038)	−0.024 (−0.034–−0.013)	<0.0001
	TGHDL	−0.161 (−0.200–−0.122)	−0.044 (−0.055–−0.034)	<0.0001
MUFA	TC, mmol/L	−0.005 (−0.013–0.003)	−0.008 (−0.021–0.005)	0.196
	LDL-c, mmol/L	−0.012 (−0.018–−0.006)	−0.023 (−0.034–−0.012)	0.001
	HDL-c, mmol/L	0.008 (0.006–0.010)	0.037 (0.028–0.046)	<0.0001
	TG, mmol/L	0.009 (−0.001–0.019)	0.013 (−0.001–0.027)	0.043
	TCHDL	−0.027 (−0.037–−0.017)	−0.038 (−0.052–−0.024)	<0.0001
	TGHDL	−0.001 (−0.013–0.011)	−0.001 (−0.014–0.012)	0.803
Animal MUFA	TC, mmol/L	0.105 (0.095–0.115)	0.111 (0.101–0.122)	<0.0001
	LDL-c, mmol/L	0.083 (0.073–0.093)	0.1 (0.088–0.112)	<0.0001
	HDL-c, mmol/L	0.009 (0.005–0.0129)	0.027 (0.015–0.038)	<0.0001
	TG, mmol/L	0.043 (0.031–0.055)	0.039 (0.028–0.049)	<0.0001
	TCHDL	0.058 (0.046–0.070)	0.051 (0.041–0.062)	<0.0001
	TGHDL	0.032 (0.016–0.048)	0.022 (0.011–0.033)	0.000
Plant MUFA	TC, mmol/L	−0.057 (−0.064–−0.049)	−0.08 (−0.091–−0.069)	<0.0001
	LDL-c, mmol/L	−0.053 (−0.059–−0.047)	−0.084 (−0.094–−0.075)	<0.0001
	HDL-c, mmol/L	0.003 (0.001–0.005)	0.012 (0.004–0.019)	0.016
	TG, mmol/L	−0.012 (−0.022–−0.002)	−0.014 (−0.026–−0.003)	0.007
	TCHDL	−0.055 (−0.063–−0.047)	−0.065 (−0.074–−0.055)	<0.0001
	TGHDL	−0.017 (−0.029–−0.005)	−0.016 (−0.026–−0.005)	0.004
PUFA	TC, mmol/L	−0.025 (−0.035–−0.015)	−0.030 (−0.042–−0.018)	<0.0001
	LDL-c, mmol/L	−0.018 (−0.026–−0.010)	−0.025 (−0.036–−0.014)	<0.0001
	HDL-c, mmol/L	0.000 (−0.004–0.004)	0.000 (−0.013–0.013)	0.863
	TG, mmol/L	−0.043 (−0.055–−0.031)	−0.044 (−0.056–−0.032)	<0.0001
	TCHDL	−0.020 (−0.030–−0.010)	−0.020 (−0.030–−0.010)	0.000
	TGHDL	−0.040 (−0.054–−0.026)	−0.032 (−0.043–−0.021)	<0.0001
Animal PUFA	TC, mmol/L	0.422 (0.377–0.467)	0.102 (0.091–0.112)	<0.0001

	LDL-c, mmol/L	0.329 (0.290–0.368)	0.090 (0.079–0.101)	<0.0001
	HDL-c, mmol/L	0.043 (0.027–0.059)	0.029 (0.018–0.039)	<0.0001
	TG, mmol/L	0.145 (0.092–0.198)	0.03 (0.019–0.041)	<0.0001
	TCHDL	0.217 (0.164–0.270)	0.044 (0.033–0.054)	<0.0001
	TGHDL	0.106 (0.037–0.175)	0.017 (0.006–0.028)	0.003
Plant PUFA	TC, mmol/L	−0.038 (−0.046–−0.030)	−0.047 (−0.056–−0.037)	<0.0001
	LDL-c, mmol/L	−0.029 (−0.037–−0.021)	−0.040 (−0.051–−0.030)	<0.0001
	HDL-c, mmol/L	−0.001 (−0.005–0.003)	−0.003 (−0.017–0.010)	0.410
	TG, mmol/L	−0.044 (−0.054–−0.034)	−0.046 (−0.056–−0.036)	<0.0001
	TCHDL	−0.027 (−0.037–−0.017)	−0.028 (−0.038–−0.018)	<0.0001
	TGHDL	−0.04 (−0.054–−0.026)	−0.032 (−0.043–−0.021)	<0.0001
Animal fat	TC, mmol/L	0.045 (0.041–0.049)	0.115 (0.105–0.125)	<0.0001
	LDL-c, mmol/L	0.035 (0.031–0.039)	0.102 (0.09–0.113)	<0.0001
	HDL-c, mmol/L	0.004 (0.002–0.006)	0.029 (0.015–0.043)	<0.0001
	TG, mmol/L	0.017 (0.011–0.029)	0.037 (0.024–0.050)	<0.0001
	TCHDL	0.025 (0.019–0.031)	0.054 (0.041–0.066)	<0.0001
	TGHDL	0.012 (0.006–0.018)	0.020 (0.010–0.030)	0.001
Plant fat	TC, mmol/L	−0.025 (−0.029–−0.021)	−0.07 (−0.081–−0.059)	<0.0001
	LDL-c, mmol/L	−0.021 (−0.025–−0.017)	−0.067 (−0.079–−0.054)	<0.0001
	HDL-c, mmol/L	0.001 (−0.001–0.003)	0.008 (−0.008–0.023)	0.222
	TG, mmol/L	−0.017 (−0.021–−0.013)	−0.041 (−0.05–−0.031)	<0.0001
	TCHDL	−0.022 (−0.026–−0.018)	−0.052 (−0.061–−0.042)	<0.0001
	TGHDL	−0.017 (−0.023–−0.011)	−0.031 (−0.042–−0.021)	<0.0001

* Adjusted for total energy, dietary fiber (energy adjusted), dietary cholesterol (energy adjusted), vegetables (energy adjusted), fruits (energy adjusted), age, sex, north/south region, urban/rural location, education (categorical), smoking (categorical by status and cigarettes), drinking status (categorical by grams), BMI (continuous), physical activity (continuous), sleep time (continuous), diagnosed hypertension (yes/no), and family history (yes/no). Abbreviation: CI, confidence interval; TC, total cholesterol; LDL-c, low-density lipoprotein cholesterol; HDL-c, high-density lipoprotein cholesterol; TG, triglyceride; TCHDL, TC-to HDL-c ratio; TGHDL, TG to HDL-c ratio.

Table S5. Blood lipid levels (TC, LDL-c, HDL-c, TCHDL, TG, TGHDL) by quintiles of various dietary fats intake (n = 39,115) *.

	Quintiles	TC, mmol/L	LDL, mmol/L	HDL, mmol/L	TG, mmol/L	TCHDL	TGHDL
Total fat	Q1	4.77	2.93	1.30	1.65	3.91	1.53
	Q2	4.81	2.96	1.31	1.63	3.90	1.48
	Q3	4.83	2.97	1.32	1.61	3.90	1.46
	Q4	4.84	2.97	1.33	1.62	3.89	1.47
	Q5	4.84	2.98	1.33	1.63	3.90	1.48
p		<0.0001	0.002	<0.0001	0.144	0.731	0.032
$p_{\text{-trend}}$		<0.0001	0.0002	<0.0001	0.181	0.274	0.033
Animal fat	Q1	4.66	2.83	1.30	1.57	3.81	1.43
	Q2	4.76	2.91	1.31	1.61	3.86	1.46
	Q3	4.81	2.96	1.32	1.62	3.89	1.47
	Q4	4.86	2.99	1.32	1.65	3.92	1.51
	Q5	4.95	3.07	1.33	1.67	3.97	1.51
p		<0.0001	<0.0001	<0.0001	<0.0001	<0.0001	0.003
$p_{\text{-trend}}$		<0.0001	<0.0001	<0.0001	<0.0001	<0.0001	0.0001
Plant fat	Q1	4.91	3.04	1.32	1.70	3.99	1.56
	Q2	4.83	2.97	1.32	1.62	3.91	1.47
	Q3	4.80	2.95	1.32	1.60	3.87	1.46
	Q4	4.77	2.93	1.32	1.59	3.86	1.44
	Q5	4.74	2.89	1.32	1.58	3.82	1.44
p		<0.0001	<0.0001	0.643	<0.0001	<0.0001	<0.0001
$p_{\text{-trend}}$		<0.0001	<0.0001	0.192	<0.0001	<0.0001	<0.0001
SFA	Q1	4.66	2.83	1.29	1.64	3.84	1.51
	Q2	4.75	2.90	1.31	1.64	3.87	1.51
	Q3	4.81	2.96	1.32	1.60	3.88	1.44
	Q4	4.88	3.01	1.33	1.61	3.90	1.45
	Q5	4.93	3.05	1.33	1.65	3.97	1.50
p		<0.0001	<0.0001	<0.0001	0.008	<0.0001	0.005
$p_{\text{-trend}}$		<0.0001	<0.0001	<0.0001	0.905	<0.0001	0.13
Animal SFA	Q1	4.64	2.82	1.30	1.56	3.80	1.43
	Q2	4.75	2.91	1.31	1.61	3.87	1.47
	Q3	4.79	2.94	1.32	1.61	3.88	1.46
	Q4	4.86	3.00	1.32	1.64	3.92	1.50
	Q5	4.94	3.06	1.33	1.67	3.97	1.52
p		<0.0001	<0.0001	<0.0001	<0.0001	<0.0001	0.008
$p_{\text{-trend}}$		<0.0001	<0.0001	<0.0001	<0.0001	<0.0001	0.001
Plant SFA	Q1	4.88	3.00	1.31	1.72	3.96	1.58
	Q2	4.79	2.94	1.31	1.64	3.89	1.50
	Q3	4.79	2.94	1.32	1.62	3.87	1.47
	Q4	4.81	2.96	1.32	1.58	3.88	1.43
	Q5	4.81	2.96	1.32	1.54	3.87	1.39
p		<0.0001	<0.0001	0.07	<0.0001	<0.0001	<0.0001
$p_{\text{-trend}}$		<0.0001	0.096	0.009	<0.0001	<0.0001	<0.0001
MUFA	Q1	4.80	2.96	1.30	1.64	3.95	1.53
	Q2	4.82	2.97	1.31	1.61	3.91	1.47
	Q3	4.85	2.98	1.33	1.59	3.89	1.43
	Q4	4.84	2.98	1.33	1.63	3.90	1.48
	Q5	4.79	2.92	1.33	1.67	3.85	1.51
p		<0.0001	<0.0001	<0.0001	<0.0001	<0.0001	0.001

$p_{\text{-trend}}$		0.89	0.034	<0.0001	0.035	<0.0001	0.896
Animal MUFA	Q1	4.67	2.84	1.30	1.57	3.82	1.44
	Q2	4.75	2.91	1.31	1.61	3.87	1.47
	Q3	4.81	2.96	1.32	1.62	3.89	1.48
	Q4	4.85	2.99	1.32	1.64	3.91	1.49
	Q5	4.96	3.08	1.33	1.68	3.98	1.52
p		<0.0001	<0.0001	<0.0001	<0.0001	<0.0001	0.015
$p_{\text{-trend}}$		<0.0001	<0.0001	<0.0001	<0.0001	<0.0001	0.001
Plant MUFA	Q1	4.91	3.03	1.31	1.70	4.00	1.57
	Q2	4.84	2.99	1.31	1.60	3.92	1.46
	Q3	4.82	2.97	1.32	1.59	3.89	1.44
	Q4	4.77	2.92	1.33	1.58	3.82	1.42
	Q5	4.69	2.84	1.32	1.64	3.79	1.49
p		<0.0001	<0.0001	0.016	<0.0000	<0.0001	<0.0001
$p_{\text{-trend}}$		<0.0001	<0.0001	0.005	0.001	<0.0001	<0.0001
PUFA	Q1	4.86	2.99	1.31	1.70	3.94	1.55
	Q2	4.82	2.96	1.32	1.64	3.90	1.49
	Q3	4.82	2.97	1.32	1.61	3.89	1.47
	Q4	4.80	2.95	1.32	1.58	3.86	1.43
	Q5	4.78	2.93	1.32	1.56	3.87	1.42
p		<0.0001	0.0001	0.129	<0.0001	<0.0001	<0.0001
$p_{\text{-trend}}$		<0.0001	<0.0002	0.130	<0.0001	<0.0001	<0.0001

* Adjusted for total energy, dietary fiber (energy adjusted), dietary cholesterol (energy adjusted), vegetables (energy adjusted), fruits (energy adjusted), age, sex, north/south region, urban/rural location, education (categorical), smoking (categorical by status and cigarettes), drinking status (categorical by grams), BMI (continuous), physical activity (continuous), sleep time (continuous), diagnosed hypertension (yes/no), and family history (yes/no).

Table S6. Beta coefficient (95% CI) for change in lipid biomarkers for per 11 g increment of animal/plant saturated fats, animal monounsaturated fats, and polyunsaturated fats, stratified by intake levels *.

**Fats**	**Lipid markers**	**<Median, E%**	**≥Median, E%**	***p*-interaction**
Animal SFA	TC, mmol/L	0.173 (0.147–0.198)	0.104 (0.090–0.118)	<0.0001
	LDL-c, mmol/L	0.129 (0.105–0.152)	0.086 (0.072–0.100)	<0.0001
	HDL-c, mmol/L	0.023 (0.013–0.033)	0.004 (-0.002–0.010)	<0.0001
	TG, mmol/L	0.058(0.027–0.089)	0.046 (0.028–0.064)	0.963
	TCHDL	0.083 (0.055–0.112)	0.066 (0.048–0.083)	0.586
	TGHDL	0.036 (-0.005–0.077)	0.035 (0.013–0.057)	0.462
Plant SFA	TC, mmol/L	−0.035 (−0.088–0.018)	−0.101 (−0.134–0.068)	0.070
	LDL-c, mmol/L	−0.008 (−0.055–0.039)	−0.07 (−0.099–0.041)	0.056
	HDL-c, mmol/L	0.011 (−0.007–0.029)	−0.006 (−0.018–0.006)	0.296
	TG, mmol/L	−0.228 (−0.291–−0.165)	−0.148 (−0.187–−0.109)	0.048
	TCHDL	−0.092 (−0.153–−0.031)	−0.065 (−0.102–0.028)	0.647
	TGHDL	−0.256 (−0.336–−0.176)	−0.129 (−0.180–−0.078)	0.017
Animal MUFA	TC, mmol/L	0.151 (0.127–0.175)	0.101 (0.087–0.115)	<0.0001
	LDL-c, mmol/L	0.115 (0.093–0.136)	0.082 (0.070–0.094)	0.000
	HDL-c, mmol/L	0.018 (0.010–0.026)	0.005 (0.001–0.009)	<0.0001
	TG, mmol/L	0.065 (0.038–0.092)	0.046 (0.030–0.062)	0.687
	TCHDL	0.075 (0.048–0.102)	0.064 (0.048–0.080)	0.908
	TGHDL	0.045 (0.008–0.082)	0.036 (0.016-0.056)	0.686
PUFA	TC, mmol/L	−0.044 (−0.064–−0.024)	−0.054 (−0.066–−0.042)	0.758
	LDL-c, mmol/L	−0.029 (−0.047–−0.011)	−0.04 (−0.0526–−0.028)	0.961
	HDL-c, mmol/L	−0.003(−0.011–0.005)	−0.007 (−0.011–−0.003)	0.926
	TG, mmol/L	−0.066 (−0.090–−0.042)	−0.045 (−0.059–−0.031)	0.156
	TCHDL	−0.030 (−0.054–−0.006)	−0.023 (−0.037–−0.009)	0.790
	TGHDL	−0.061 (−0.092–−0.030)	−0.036 (−0.056–−0.016)	0.278

* Stratified by median of total fat intake (68.7 g, corresponding to 34.9% of total energy).

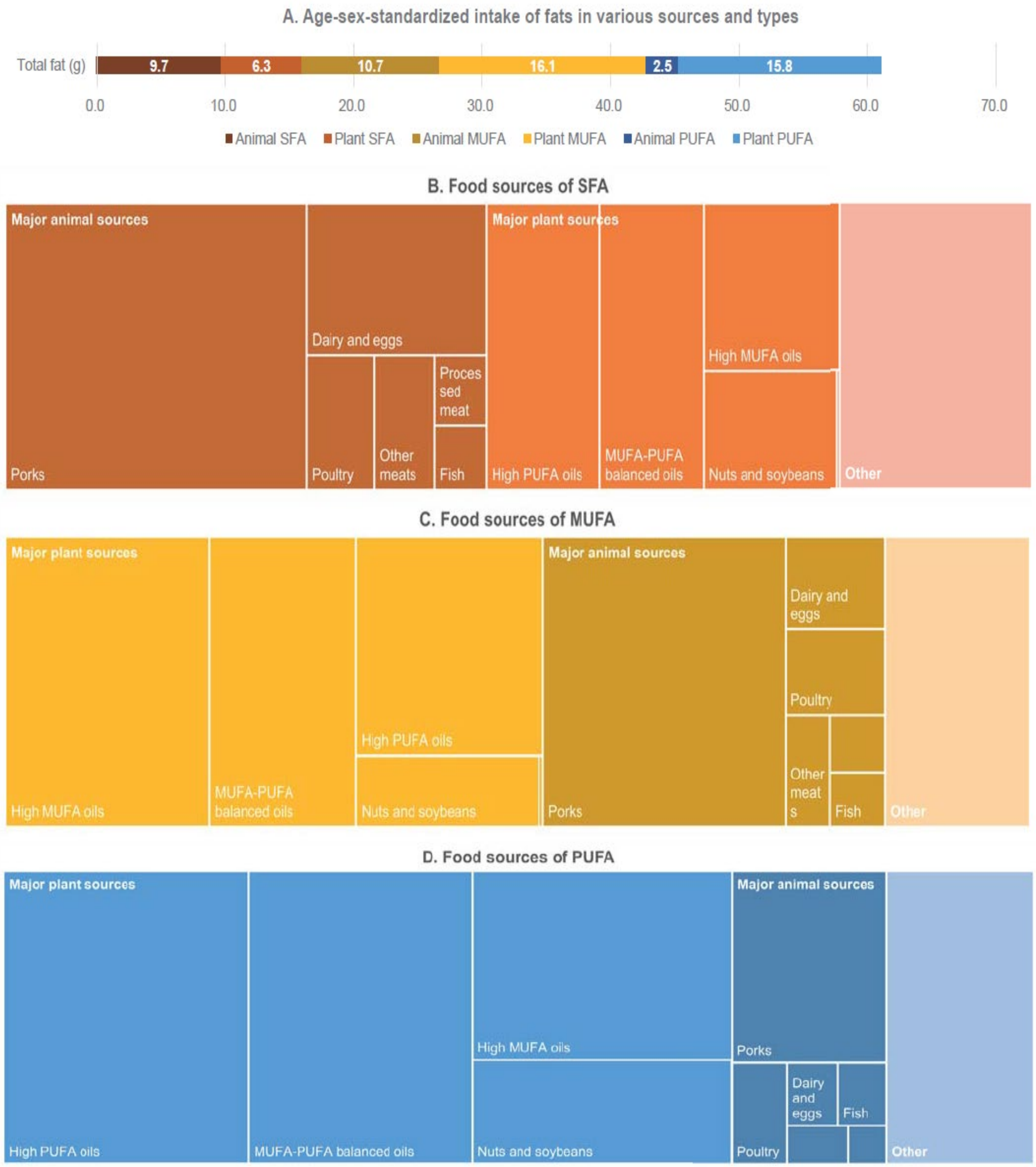

Figure S1. Quantity and food sources of dietary fats in various types in survey population. The area in the box represents the proportion of fat from specific food sources in corresponding type of fat. Abbreviation: MUFA, monounsaturated fatty acids; PUFA, polyunsaturated fatty acids; SFA, saturated fatty acids.

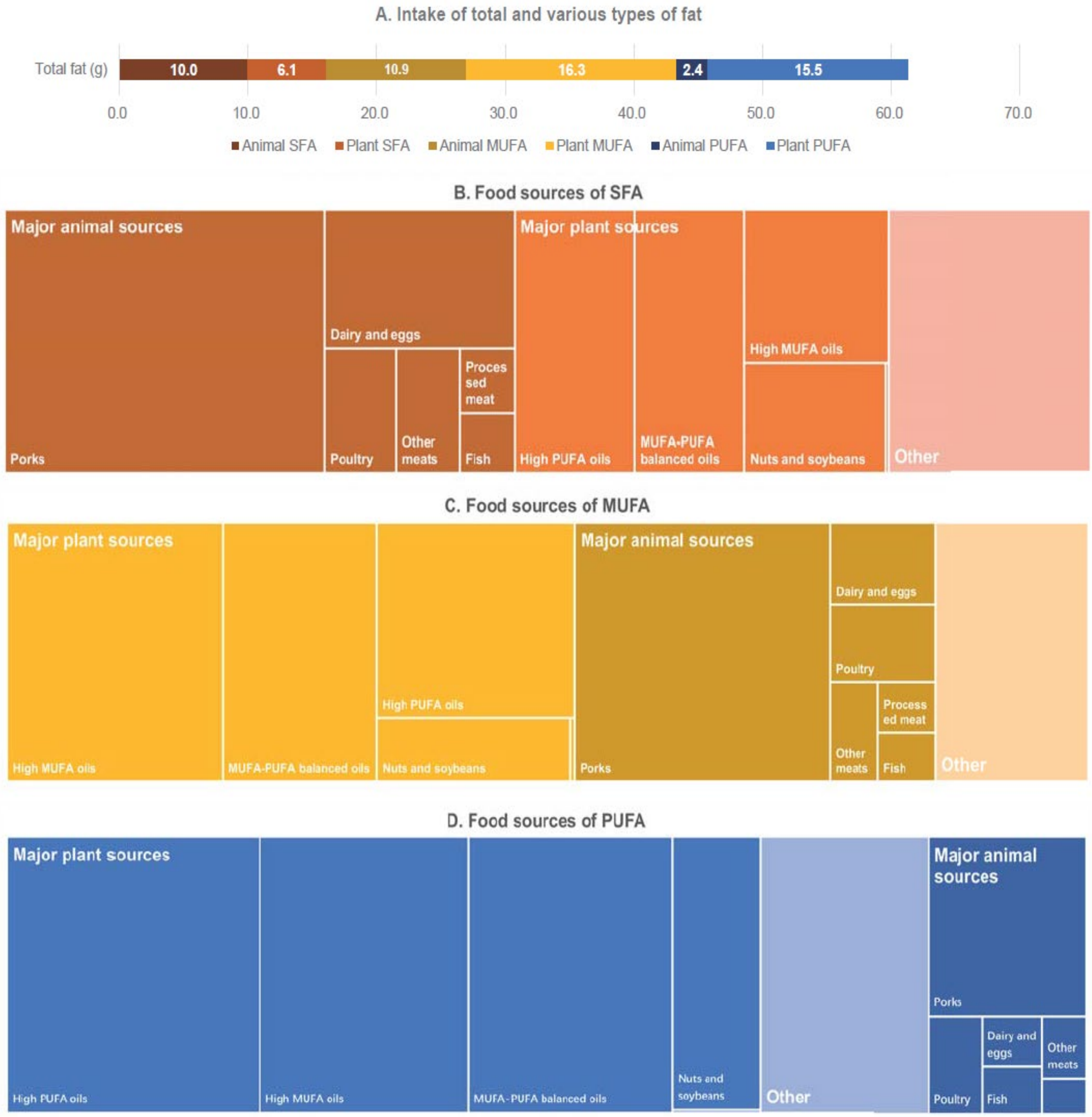

Figure S2. Quantity and food sources of dietary fats in various types in selected sample (n = 39,115). The area in the box represents the proportion of fat from specific food sources in corresponding type of fat.
